# Blockade of TASK-1 Channel Improves the Efficacy of Levetiracetam in Chronically Epileptic Rats

**DOI:** 10.3390/biomedicines10040787

**Published:** 2022-03-28

**Authors:** Ji-Eun Kim, Tae-Cheon Kang

**Affiliations:** Department of Anatomy and Neurobiology and Institute of Epilepsy Research, College of Medicine, Hallym University, Chuncheon 24252, Korea; jieunkim@hallym.ac.kr

**Keywords:** astrocyte, intractable epilepsy, ML365, pharmacoresistant epilepsy, refractory seizure

## Abstract

Tandem of P domains in a weak inwardly rectifying K^+^ channel (TWIK)-related acid sensitive K^+^-1 channel (TASK-1) is an outwardly rectifying K^+^ channel that acts in response to extracellular pH. TASK-1 is upregulated in the astrocytes (particularly in the CA1 region) of the hippocampi of patients with temporal lobe epilepsy and chronically epilepsy rats. Since levetiracetam (LEV) is an effective inhibitor for carbonic anhydrase, which has a pivotal role in buffering of extracellular pH, it is likely that the anti-epileptic action of LEV may be relevant to TASK-1 inhibition, which remains to be elusive. In the present study, we found that LEV diminished the upregulated TASK-1 expression in the CA1 astrocytes of responders (whose seizure activities were responsive to LEV), but not non-responders (whose seizure activities were not controlled by LEV) in chronically epileptic rats. ML365 (a selective TASK-1 inhibitor) only reduced seizure duration in LEV non-responders, concomitant with astroglial TASK-1 downregulation. Furthermore, ML365 co-treatment with LEV decreased the duration, frequency and severity of spontaneous seizures in non-responders to LEV. To the best of our knowledge, our findings suggest, for the first time, that the up-regulation of TASK-1 expression in CA1 astrocytes may be involved in refractory seizures in response to LEV. This may be a potential target to improve responsiveness to LEV.

## 1. Introduction

Epilepsy is a chronic neurological disease that is characterized by the presence of spontaneous aberrant neuronal discharges which manifest as seizures. The causes of epileptic seizure generation (ictogenesis) are imbalance of excitatory/inhibitory transmissions, channelopathies, neuroinflammation, and aberrant synaptic plasticity [1,2,3,4]. Since shifts in activity-dependent intracellular and extracellular pH regulate the initiation and cessation of seizure activity [5,6,7], impaired acid-base balance also contributes to an augmented capability to generate epileptic discharges. Indeed, recurrent epileptiform activity results in biphasic pH shifts, consisting of an initial extracellular alkalinization followed by a slower acidification in the CA1 region of the hippocampus [7]. Extracellular alkalinization decreases the inhibitory conductance through γ-aminobutyric acid type A receptor (GABA_A_ receptor) [8] and increases the N-methyl-D-aspartate (NMDA) receptor-mediated excitatory current [9,10]. Thus, activity-dependent extracellular alkaline shifts initiate the seizure activity, while extracellular acidification terminates the epileptiform activity [6,7].

Astrocytes play an important role in the redistribution of K^+^ in extracellular space (K^+^ buffering) that is involved in the control of resting membrane potential and neuronal firing. When K^+^ buffering becomes hindered, an accumulation of extracellular K^+^ leads to hyperexcitability of neurons by inhibiting K^+^ efflux from neurons during repolarization. Interestingly, astrocytes in the CA1 region (CA1 astrocytes) have lost barium (Ba^2+^)-sensitive K^+^ buffering in the epileptic hippocampi of humans and rats, unlike those in the dentate gyrus [11,12]. Furthermore, the ratio of inward-to-outward K^+^ conductance in astrocytes is significantly lower in the hippocampus of temporal lobe epilepsy (TLE) patients [13]. Therefore, the dysfunction of astrocyte-mediated extracellular K^+^ homeostasis is an important factor in the pathogenesis of epilepsy.

Tandem of P domains in a weak inwardly rectifying K^+^ channel (TWIK)-related acid sensitive K^+^-1 channel (TASK-1) is an outwardly rectifying K^+^ channel that acts in response to extracellular pH. Low extracellular pH (6.0–6.4) completely inhibits TASK-1 current, whereas high extracellular pH (7.2–8.2) potentiates it [14,15]. TASK-1 is upregulated in astrocytes (particularly in the CA1 region) of the hippocampi of TLE patients and chronically epileptic rats [16,17]. Furthermore, conventional antiepileptic drugs (AEDs, currently termed as anti-seizure medication (ASM)) reduce astroglial TASK-1 expression in seizure-prone gerbils (a genetic epilepsy model) [18]. Thus, it is plausible that TASK-1 upregulation can contribute to seizure activity by increasing astroglial K^+^ outward rectification in response to extracellular alkalinization.

More than ~10–30% of TLE patients show intractable seizures that are uncontrolled by AEDs [19]. Therefore, pharmacoresistances to AEDs are a major clinical problem in the medication of TLE patients. The anti-epileptic properties of levetiracetam (LEV, 2*S*-(oxo-1-pyrrolidinyl)butanamide, Keppra^®^) are relevant to the high-affinity binding to synaptic vesicle protein 2A (SV2A) that may affect presynaptic neurotransmitter release. Although LEV treatment often results in seizure-free conditions in TLE patients who show refractory seizures to other AEDs, LEV does not mitigate spontaneous seizures in approximately 30% of TLE patients at the beginning of the pharmacotherapy [20]. Similar to the case of TLE patients, ~25–40% of pilocarpine-induced chronically epileptic rats do not show a significant response to LEV [21,22]. On the other hand, LEV also leads to a pH shift by inhibiting the transmembrane HCO_3_^−^-mediated acid extrusion and carbonic anhydrase, like other AEDs [23,24,25,26,27]. Thus, it is likely that the anticonvulsive potency of LEV may be closely relevant to the induction of extracellular acidification, which would inhibit the upregulated TASK-1 in the epileptic hippocampus. In the present study, therefore, we performed a comparative analysis of TASK-1 expression in the hippocampi of responders (whose seizure activities were responsive to LEV) and non-responders (whose seizure activities were uncontrolled by LEV) in chronically epileptic rats, and validated the effect of ML365 (a selective TASK-1 inhibitor) co-treatment with LEV on refractory seizures in response to LEV to extend our understanding of the underlying mechanisms of pharmacoresistant epilepsy.

## 2. Materials and Methods

### 2.1. Experimental Animals and Chemicals

In the present study, we used male Sprague-Dawley (SD) rats (7 weeks old). Rats were housed in a controlled environment at a humidity of 55 ± 5% and a temperature of 22 ± 2 °C on a 12 h light/dark cycle and provided with food and water ad libitum [22,28,29]. All animal studies were performed in accordance with protocols approved by the Institutional Animal Care and Use Committee of Hallym University (No. Hallym 2018-2, 26 April 2018; No. Hallym 2018-21, 8 June 2018; and Hallym 2021-3, 27 April 2021). All reagents, unless otherwise noted, were obtained from Sigma-Aldrich (St. Louis, MO, USA).

### 2.2. Generation of Chronically Epileptic Rats

To generate chronically epileptic rats, we applied the status epilepticus (SE) model. For SE induction, animals were treated with LiCl (127 mg/kg) via intraperitoneal injection (i.p.) 24 h prior to pilocarpine treatment. Animals were given atropine methylbromide (5 mg/kg i.p.) 20 min before pilocarpine (30 mg/kg). Two h after SE onset, all rats received diazepam (Hoffman la Roche, Neuilly sur-Seine, France; 10 mg/kg, i.p.) to cease SE and was repeated as needed. The control rats were treated with the same volume of saline in place of pilocarpine. SE-experiencing rats were video monitored 8 h a day to select for the chronically epileptic rats showing the occurrence of spontaneous seizures (Racine’s scale ≥ 3 more than once) [22,28,29,30].

### 2.3. Electrode Implantation and ML365 Infusion

The control and epilepsy rats were anesthetized with isoflurane anesthesia (3% induction, 1.5–2% for surgery, and 1.5% maintenance in a 65:35 mixture of N_2_O:O_2_) and placed in a stereotaxic frame. Thereafter, a monopolar stainless-steel electrode (#MS303-1-AIU-SPC, diameter 0.01 inch, Plastics One, Roanoke, VA, USA) was implanted in the right hippocampus at the following coordinates: 3.8 mm posterior, 2.0 mm lateral and −2.6 mm depth to bregma. A brain infusion kit 1 (Alzet, Cupertino, CA, USA) was also inserted into some animals for the infusion of vehicle or ML365 (a specific TASK-1 inhibitor, 400 nM [31]) into the right lateral ventricle at the following coordinates: 1 mm posterior, 1.5 mm lateral and 3.5 mm depth to the bregma. The electrode and brain infusion kits were secured to the exposed skull with dental acrylic [22,28,29].

### 2.4. Drug Trial Protocols

**Figure 1:**
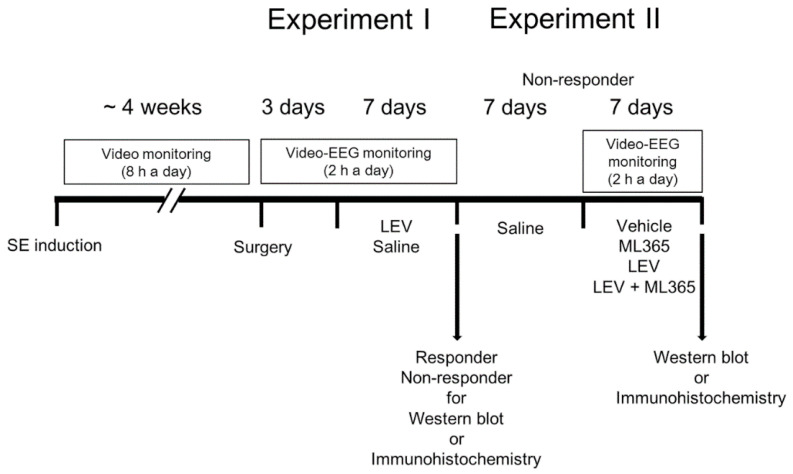
Scheme of the experimental design in the present study.

Figure 1 illustrates the experimental design in the present study, which is a modified drug trial methodology based on our previous studies [22,28,29].

#### 2.4.1. Experiment I

Baseline seizure activity in chronically epileptic rats were recorded over a 3-day period. Electroencephalographic (EEG) signals were acquired with a DAM 80 differential amplifier (0.1–1000 Hz bandpass; World Precision Instruments, Sarasota, FL, USA) 2 h a day at the same time over a 7-day period. Thereafter, animals received LEV (500 mg/kg, i.p., UCB Korea, Seoul, Korea) or saline (vehicle) once a day (at 6:00 p.m.) over a 7-day period [22,28,29]. The EEG data were digitized and analyzed using a LabChart Pro v7 (ADInstruments, Bella Vista, New South Wales, Australia). Racine’s scale was applied to quantify behavioral seizure severity, as aforementioned. Animals whose seizure frequency was unaffected by LEV during recording, as compared to the pre-treatment stage, were defined as non-responders (Figure 1).

#### 2.4.2. Experiment II

After saline treatment over a 7-day period, non-responders in experiment I were infused vehicle or ML365 (400 nM [31]) by connecting Alzet 1007D osmotic pump (Alzet, Cupertino, CA, USA). The pump was inserted into a subcutaneous pocket in the dorsal region. Some animals were also administered LEV (500 mg/kg, i.p., UCB Korea, Seoul, Korea) once a day.

### 2.5. Western Blot

After recording (18 h after the last drug treatment), animals were sacrificed by decapitation. Thereafter, the hippocampi were rapidly dissected and homogenized in lysis buffer. The lysis buffer contained protease inhibitor cocktail (Roche Applied Sciences, Branford, CT, USA) and phosphatase inhibitor cocktail (PhosSTOP^®^, Roche Applied Science, Branford, CT, USA). The protein concentration was determined using a Micro BCA Protein Assay Kit (Pierce Chemical, Rockford, IL, USA). An equal amount (10 μg each) was loaded on a bis-tris sodium dodecyl sulfate-polyacrylamide gel (SDS-PAGE). The proteins were separated by electrophoresis and transferred to membranes. The membranes were blocked with tris-buffered saline (TBS; in mM 10 Tris, 150 NaCl, pH 7.5, and 0.05% Tween 20) containing 2% bovine serum albumin and then incubated with primary antibodies (Table 1) overnight at 4 °C. The proteins were visualized using an electrochemiluminescence (ECL) Western Blotting System (GE Healthcare Korea, Seoul, Korea). For data normalization, β-actin was used as an internal reference. An ImageQuant LAS4000 system (GE Healthcare Korea, Seoul, Korea) was used to detect and quantify the Western blot data [22,28,29].

### 2.6. Immunohistochemistry

Animals were transcardially perfused and fixed with 4% paraformaldehyde under deep anesthesia with urethane (1.5 g/kg, i.p.). The brains were then removed and postfixed in the same fixative overnight and left in 30% sucrose in phosphate buffer (PB) until sunk. Coronal sections (30 μm) of the brain samples were cut using a cryostat. Sections were placed in a plate, rinsed with phosphate-buffered saline (PBS) for over 10 min, and subsequently blocked for 30 min at room temperature in 10% goat serum (Vector, Burlingame, CA, USA). After blocking, samples were incubated with primary antibodies overnight at 4 °C. Sections were then washed for over 10 min three times with PBS and incubated with appropriate secondary antibodies (1:200, Vector, Burlingame, CA, USA) for 1 h at room temperature. After washing, sections were mounted in Vectashield mounting media with 4′,6-diamidino-2-phenyulindole (DAPI, Vector, Burlingame, CA, USA). The brain sections incubated with either preimmune serum (for GFAP) or the primary antibody reacted with the control peptide (for TASK-1) were used as negative controls [16]. Immunoreaction was observed using an Axio Scope microscope (Carl Zeiss Korea, Seoul, Korea). Five hippocampal sections from each animal were randomly captured and the areas of interest (1 × 10^5^ μm^2^) were selected from the stratum radiatum and the stratum pyramidale of the CA1 region. Thereafter, fluorescent intensity and the number of double-stained cells was measured using AxioVision Rel. 4.8 and ImageJ software. The quantification of TASK-1 fluorescent intensity and double-stained cells was performed in the left hippocampus to avoid the interfering effect of reactive astrogliosis induced by electrode implantation. The investigators were blinded to experimental groups in performing morphological analysis and immunohistochemical experiments [28,29].

### 2.7. Data Analysis

In the present study, the effects of each compound on seizures were analyzed based on the following seizure parameters: Seizure frequency was number of seizures in each animal during the 2 h recording. Seizure duration was the overall time spent in convulsive and non-convulsive seizures in each animal during the 2 h recording. Seizure severity was the behavioral seizure score in each animal during the 2 h recording. Total seizure frequency was the total seizure occurrence (number of seizures) in each animal over a 7-day period. Total seizure duration was the overall time spent in convulsive and non-convulsive seizures in each animal over a 7-day period. Average seizure severity was the average behavioral seizure core in each animal over a 7-day period. Seizure parameters were assessed by different investigators who were blind to the classification of animal groups and treatments. All data are presented as the means ± standard deviation (SD) or standard error of mean (SEM). After the Shapiro–Wilk *W*-test was used to evaluate the values on normality, the Student’s *t*-test (for total seizure duration, immunohistochemistry and Western blot data), the Mann–Whitney U test (total seizure frequency and average seizure severity), a repeated measures ANOVA (seizure duration over a 7-day period), the Friedman test (seizure frequency and seizure severity over a 7-day period) and a one-way ANOVA followed by Bonferroni’s post hoc comparisons (for immunohistochemistry and Western blot data) were applied to determine the statistical significance of the data. A *p*-value less than 0.05 was considered statistically significant.

## 3. Results

### 3.1. Effects of LEV on Spontaneous Epileptic Seizures

**Figure 2:**
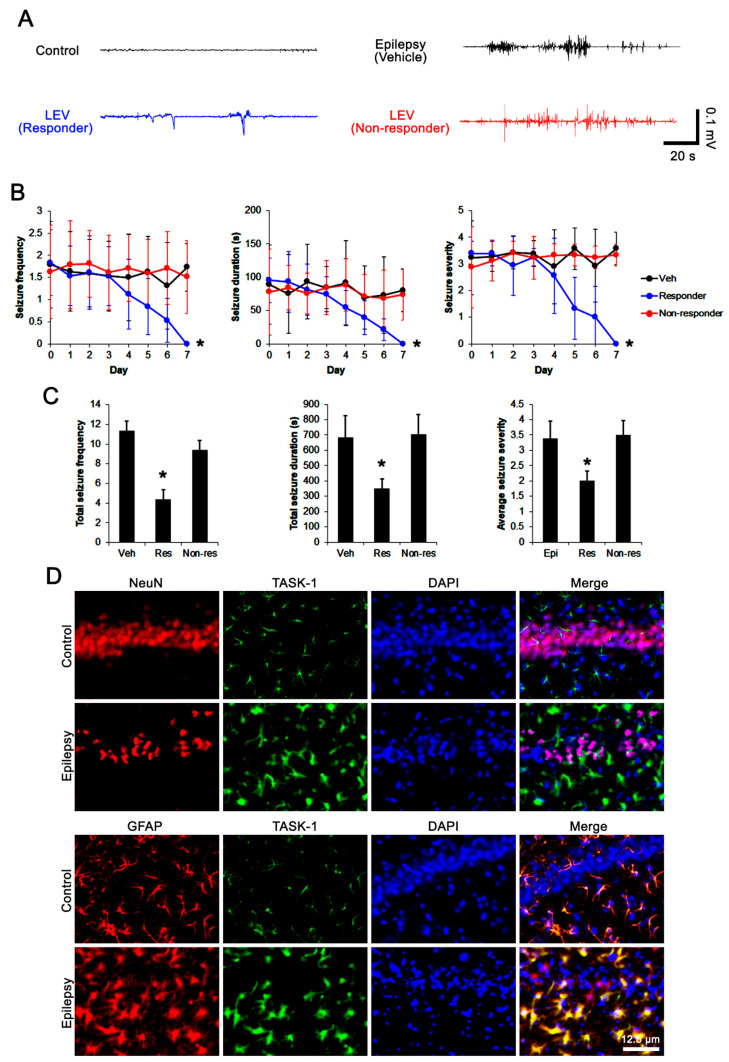
The effects of levetiracetam (LEV) on spontaneous seizure activities in chronically epileptic rats and the localization TASK-1 in the hippocampus of the control and chronically epileptic rats. LEV effectively attenuated spontaneous seizure activity on EEG in responders (**A**) at 4 days after treatment, accompanied by reductions in seizure frequency (Friedman test), seizure duration (repeated measures ANOVA), seizure severity (seizure score, Friedman test), total seizure frequency (Mann–Whitney U test), total seizure duration (Student’s *t*-test) and average behavioral seizure score (seizure severity; Mann–Whitney U test) over a 7-day period (**B**,**C**; *, *p* < 0.05 vs. vehicle (Veh)-treated animals; error bars, SD). However, the non-responders did not respond to LEV treatment (**B**,**C**). Double immunofluorescent data reveal that TASK-1 expression is observed in GFAP-positive astrocytes, but not in NeuN-positive neurons in both control and chronically epileptic rats (**D**).

First, we explored the efficacy of LEV on spontaneous seizure activity in chronically epileptic rats. In vehicle-treated epileptic rats (*n* = 14), total seizure frequency (number of seizures), total electroencephalographic (EEG) seizure duration and average seizure severity (behavioral seizure core) were 11.3 ± 2.1, 681 ± 145 s, and 3.4 ± 0.6 over a 7-day period, respectively (Figure 2A–C). About 57% of the LEV-treated group (*n* = 70) were identified as responders whose seizure frequency (*χ*^2^_(7)_ = 19.4, *p* = 0.007, Friedman test), seizure duration (*F*_(7,483)_ = 4.1, *p* < 0.001, repeated measures ANOVA), and seizure severity (*χ*^2^_(7)_ = 17.8, *p* = 0.013, Friedman test) were effectively alleviated by LEV treatment over a 7-day period (Figure 2A,B). In this group, total seizure frequency, total seizure duration and average seizure severity were also attenuated to 4.4 ± 1.2 (Z = 3.2, *p* = 0.002 vs. vehicle, Mann–Whitney U-test), 349 ± 65 s (*t*_(82)_ = 3.9, *p* = 0.002 vs. vehicle, Student *t*-test), and 2 ± 0.3 (Z = 2.4, *p* = 0.01 vs. vehicle, Mann–Whitney U test) over a 7-day period, respectively (Figure 2C). LEV did not influence seizure activity in 52 out of 122 rats (~43% in LEV-treated rats). Thus, they were identified as non-responders (Figure 2A–C).

### 3.2. Effects of LEV on TASK-1 Expression in the Epileptic Hippocampus

**Figure 3:**
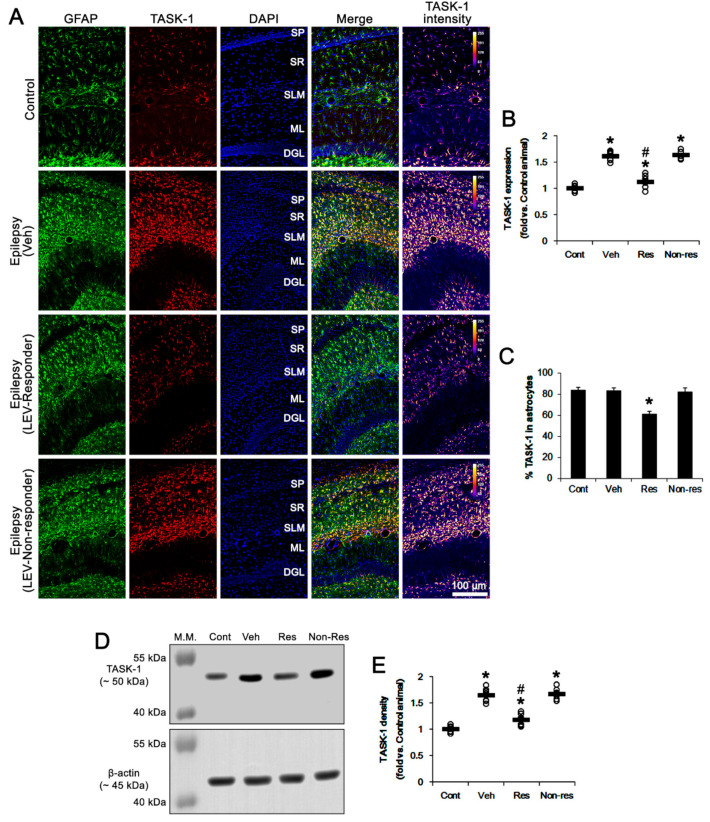
The effect of levetiracetam (LEV) on TASK-1 expression in chronically epileptic rats. As compared to control animals, TASK-1 expression is upregulated in CA1 astrocytes of epileptic rats. LEV significantly attenuated TASK-1 upregulation in responders, but not non-responders. (**A**) Representative photos of the TASK-1 expression in the hippocampus. SP, stratum pyramidale; SR, stratum radiatum; SLM, stratum lacunosum-moleculare; ML, molecular layer of the dentate gyrus; DGL, dentate granule cell layer. (**B**) Quantitative analyses of the effect of LEV on TASK-1 expression based on the immunohistochemical data (*,#, *p* < 0.05 vs. the control and vehicle (Veh)-treated animals, respectively; one-way ANOVA with *post hoc* Bonferroni’s multiple comparison, open circles indicate each individual value, horizontal bars indicate the mean value, error bars indicate the SEM). (**C**) Quantitative analyses on the effect of LEV on the fraction of TASK-1 positive astrocytes in total astrocytes (*, *p* < 0.05 vs. control animals; one-way ANOVA with post hoc Bonferroni’s multiple comparison; error bars, SEM). (**D**,**E**) Representative images for Western blot of TASK-1 protein expression in the hippocampal tissues and quantifications of TASK-1 level based on Western blot results (*,#, *p* < 0.05 vs. control and vehicle (Veh)-treated animals, respectively; one-way ANOVA with post hoc Bonferroni’s multiple comparison).

Consistent with previous studies [16,17,32], TASK-1 expression was rarely observed in CA1 neurons in both control and epileptic rats (Figure 2D). In contrast, TASK-1 expression was prominently detected in the astrocytes within the stratum radiatum and stratum lacunosum-moleculare of the CA1 region of the control rats (*n* = 7). TASK-1 expression was also detected in the astrocytes in the molecular layer and the hilus of the dentate gyrus of control rats (Figure 2D and Figure 3A). In chronically epileptic rats (*n* = 7), TASK-1 expression was obviously detected in most of the reactive CA1 astrocytes, showing hypertrophy and hyperplasia of cell bodies and processes (Figure 2D). TASK-1 expression was rarely observed in reactive astrocytes within the dentate gyrus (Figure 3A). TASK-1 fluorescent intensity was increased to 1.62-fold of control level (*t*_(12)_ = 14.4, *p* < 0.001, Student’s *t*-test, Figure 3A,B). However, the fraction of TASK-1 positive astrocytes in total astrocytes of chronically epileptic rats was similar to that in control animals (Figure 3C). As compared to the vehicle-treated animals (*n* = 7), responders to LEV (*n* = 7) showed a reduction in TASK-1 fluorescent intensity (*F*_(2,17)_ = 49.7, *p* < 0.001, one-way ANOVA), while the TASK-1 intensity in non-responders to LEV (*n* = 6) was unaffected by LEV treatment (Figure 3A,B). Thus, the fraction of TASK-1 positive astrocytes in total astrocytes was decreased in responders, as compared to the vehicle-treated animals (*F*_(2,17)_ = 5.4, *p* = 0.015, one-way ANOVA, Figure 3C). Compatible with the immunohistochemical data, the Western blot revealed that TASK-1 density was elevated to 1.65-fold of control level in chronically epileptic rats (*t*_(12)_ = 11.9, *p* < 0.001, *n* = 7, Student’s *t*-test, Figure 3C,D). In responders (*n* = 7), LEV reduced TASK-1 density in the hippocampus (*F*_(2,17)_ = 37.1, *p* < 0.001, one-way ANOVA) but not in the non-responders (*n* = 6 Figure 3C,D, Supplementary Figure S1). Together with the effects of LEV on seizure activity, these findings indicate that the upregulated TASK-1 expression in CA1 astrocytes may be relevant to spontaneous seizure activity and affect the responsiveness to LEV.

### 3.3. Effects of ML365 on the Spontaneous Seizures and TASK-1 Expression in Chronically Epileptic Rats

**Figure 4:**
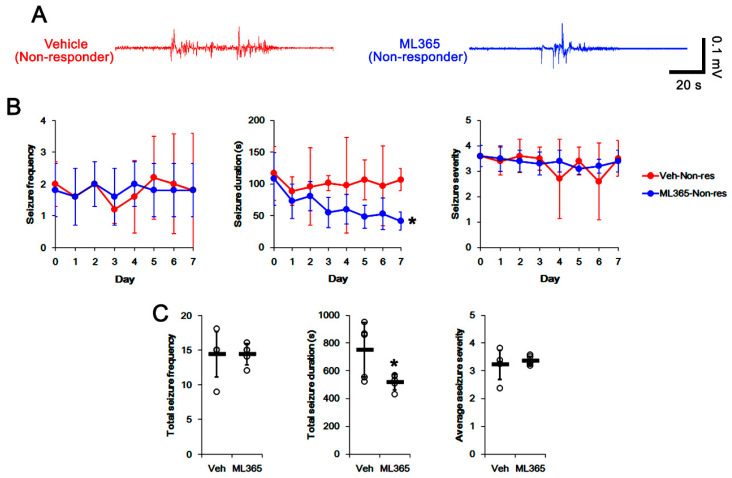
The effects of ML365 on spontaneous seizure activities in non-responders to levetiracetam (LEV). ML365 diminished only seizure duration. (**A**) Representative EEG signal in each group at 4 days after treatment. (**B**) Quantitative analyses of the effects of LEV on seizure frequency (Friedman test), seizure duration (repeated measures ANOVA) and seizure severity (seizure score, Friedman test) over a 7-day period (*, *p* < 0.05 vs. vehicle (Veh)-treated animals; error bars, SD). (**C**) Quantitative analyses of total seizure frequency (Mann–Whitney U test), total seizure duration (Student’s *t*-test) and average behavioral seizure score (seizure severity, Mann–Whitney U test) over a 7-day period (*, *p* < 0.05 vs. vehicle (Veh)-treated animals; open circles, each individual value; horizontal bars, mean value; error bars, SD).

**Figure 5:**
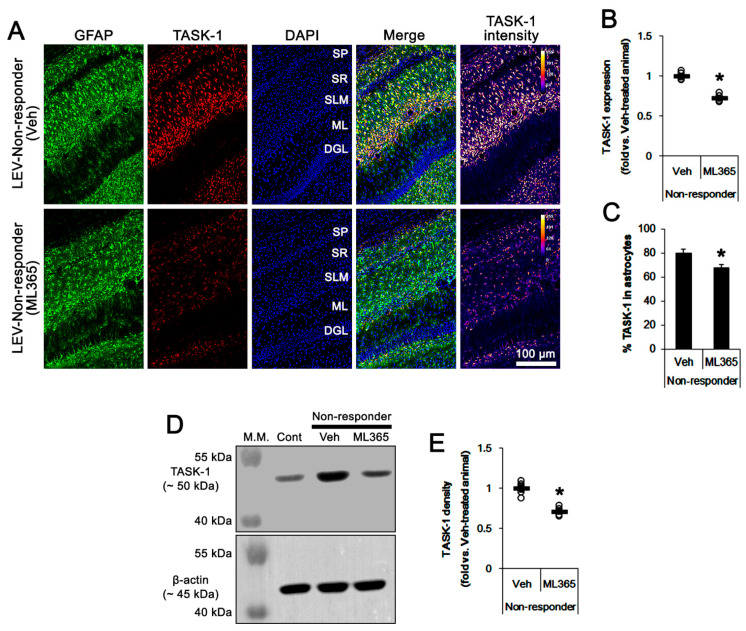
The effect of ML365 on TASK-1 expression in non-responders to levetiracetam (LEV). As compared to the vehicle (Veh), ML365 reduces TASK-1 expression in CA1 astrocytes within the hippocampus of non-responders. (**A**) Representative photos of TASK-1 expression in the hippocampus (SP, stratum pyramidale; SR, stratum radiatum; SLM, stratum lacunosum-moleculare; ML, molecular layer of the dentate gyrus; DGL, dentate granule cell layer). (**B**) Quantitative analyses of the effect of ML365 on TASK-1 expression based on immunohistochemical data. Open circles indicate each individual value. Horizontal bars indicate the mean value. Error bars indicate SEM (*, *p* < 0.05 vs. vehicle-treated animals; Student’s *t*-test). (**C**) Quantitative analyses of the effect of ML365 on the fraction of TASK-1 positive astrocytes in total astrocytes (*, *p* < 0.05 vs. control animals; Student’s *t*-test; error bars, SEM). (**D**) Representative images for Western blot of TASK-1 protein expression in the hippocampal tissues. (**E**) Quantification of TASK-1 levels based on Western blots (*, *p* < 0.05 vs. vehicle-treated animals; Student’s *t*-test).

To directly investigate the role of TASK-1 in spontaneous seizure activity, we applied ML365 (a selective TASK-1 inhibitor) to non-responders to LEV. In vehicle-treated non-responders to LEV (*n* = 10), the total seizure frequency, total EEG seizure duration and average seizure severity over a 7-day period were 14.4 ± 3.3, 751 ± 197 s, and 3.2 ± 0.5, respectively (Figure 4A–C). In ML365-treated non-responders to LEV (*n* = 10), seizure duration was gradually decreased over a 7-day period (*F*_(7,63)_ = 3.326, *p* = 0.004, repeated measures ANOVA; Figure 4A,B). In this group, the total seizure duration was also diminished to 519 ± 55 s over a 7-day period (*t*_(8)_ = 2.5, *p* = 0.035 vs. vehicle, Student’s *t*-test; Figure 4C). Furthermore, ML365 reduced the TASK-1 fluorescent intensity to 0.73-fold of the vehicle levels (*t*_(8)_ = 8.8, *p* < 0.001 vs. vehicle, Student’s *t*-test, *n* = 5). In addition, ML365 diminished the fraction of TASK-1 positive astrocytes of the total astrocytes (*t*_(8)_ = 2.7, *p* = 0.027, Student’s *t*-test, Figure 5C). Consistent with the immunohistochemical study, the Western blot data showed a reduction in TASK-1 density to 0.71-fold of the vehicle levels (*t*_(8)_ = 6.4, *p* < 0.001 vs. vehicle, Student’s *t*-test, *n* = 5) in ML365-treated non-responders (Figure 5D,E, Supplementary Figure S1). These findings indicate that TASK-1 in reactive CA1 astrocytes may be involved in the prolongation of seizure duration rather than seizure generation (ictogenesis) in non-responders to LEV. 

### 3.4. Effect of ML365 Co-Treatment on Refractory Seizures in Non-Responders to LEV

**Figure 6:**
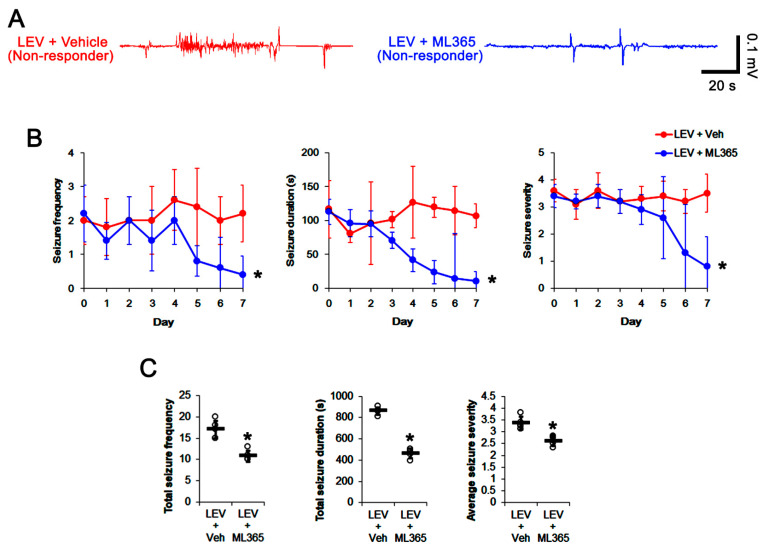
The effect of ML365 co-treatment with levetiracetam (LEV) on spontaneous seizure activities in non-responders to LEV. ML365 co-treatment improved the efficacy of LEV in non-responders. (**A**) Representative EEG signal in each group at 4 days after treatment. (**B**) Quantitative analyses of the effects of ML365 co-treatment on seizure frequency (Friedman test), seizure duration (repeated measures ANOVA) and seizure severity (seizure score, Friedman test) over a 7-day period (*, *p* < 0.05 vs. vehicle (Veh)-treated animals; error bars, SD). (**C**) Quantitative analyses of total seizure frequency (Mann–Whitney U test), total seizure duration (Student’s *t*-test) and average behavioral seizure score (seizure severity, Mann–Whitney U test) over a 7-day period (*, *p* < 0.05 vs. vehicle (Veh)-treated animals; open circles, each individual value; horizontal bars, mean value; error bars, SD).

**Figure 7:**
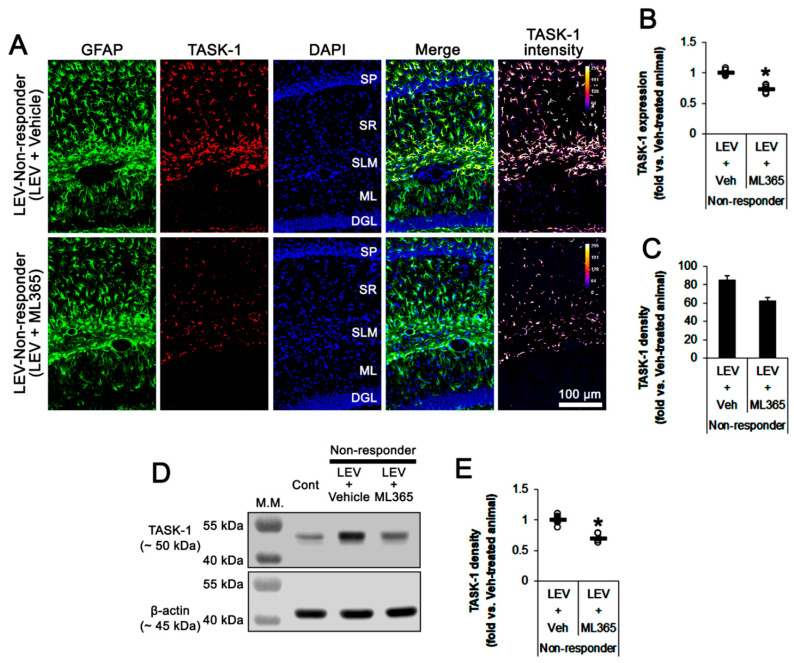
The effect of ML365 co-treatment with levetiracetam (LEV) on TASK-1 expression in non-responders to LEV. ML365 co-treatment reduced TASK-1 expression in CA1 astrocytes within the hippocampus of non-responders. (**A**) Representative photos of TASK-1 expression in the hippocampus (SP, stratum pyramidale; SR, stratum radiatum; SLM, stratum lacunosum-moleculare; ML, molecular layer of the dentate gyrus; DGL, dentate granule cell layer). (**B**) Quantitative analyses of the effect of ML365 co-treatment on TASK-1 expression based on immunohistochemical data. Open circles indicate each individual value. Horizontal bars indicate the mean value. Error bars indicate SEM (*, *p* < 0.05 vs. LEV-treated animals; Student’s *t*-test). (**C**) Quantitative analyses of the effect of ML365 co-treatment on the fraction of TASK-1 positive astrocytes in total astrocytes (*, *p* < 0.05 vs. control animals; Student’s *t*-test; error bars, SEM). (**D**) Representative images for Western blots of TASK-1 protein expression in the hippocampal tissues. (**E**) Quantifications of TASK-1 levels based on Western blot results (*, *p* < 0.05 vs. LEV-treated animals; Student’s *t*-test).

When considering the effect of ML365 on seizure activity and TASK-1 expression in CA1 astrocyte (Figure 4 and Figure 5), it is likely that ML365 co-treatment may improve the efficacy of LEV in non-responders. Thus, we validated the effects of ML365 co-treatment on intractable seizures in non-responders to LEV. As compared to vehicle co-treatment (*n* = 10), ML365 co-treatment (*n* = 10) gradually reduced seizure frequency (*χ*^2^_(7)_ = 18.9, *p* = 0.009, Friedman test), seizure duration (*F*_(7,63)_ = 25.1, *p* < 0.001, repeated measures ANOVA) and seizure severity (*χ*^2^_(7)_ = 15.6, *p* = 0.029, Friedman test) in non-responders to LEV over a 7-day period (Figure 6A,B), although it could not completely inhibit spontaneous seizure activity (Figure 6A,B). ML365 co-treatment also diminished total seizure frequency (Z = 2.7, *p* = 0.007 vs. vehicle co-treatment, Mann–Whitney U test), total seizure duration (*t*_(8)_ = 15.7, *p* < 0.001 vs. vehicle co-treatment, Student’s *t*-test) and average seizure severity (Z = 2.6, *p* = 0.009 vs. vehicle co-treatment, Mann–Whitney U test) in non-responders to LEV over a 7-day period (Figure 6C). In addition, ML365 co-treatment decreased TASK-1 fluorescent intensity to 0.73-fold of the vehicle co-treatment animal level (*t*_(8)_ = 7, *p* < 0.001 vs. vehicle co-treatment, Student’s *t*-test, *n* = 5) in non-responders. ML365 co-treatment reduced the fraction of TASK-1 positive astrocytes of the total astrocytes (*t*_(8)_ = 2.9, *p* = 0.019, Student’s *t*-test, Figure 7A–C). TASK-1 density also diminished to 0.7-fold of the vehicle co-treated animal level (*t*_(8)_ = 6, *p* < 0.001 vs. vehicle co-treatment, Student’s *t*-test, *n* = 5) in non-responders (Figure 7D,E, Supplementary Figure S1). Taken together, our findings suggest that TASK-1 inhibition by ML365 may improve the efficacy of LEV in non-responders.

## 4. Discussion

The major findings of the present study are that (1) LEV reduced TASK-1 expression in reactive CA1 astrocytes of responders, (2) TASK-1 inhibition by ML365 shortened seizure duration in non-responders, and (3) ML365 co-treatment with LEV effectively attenuated seizure activity in non-responders.

In the present study, 43% of chronically epileptic rats did not show a significant response to LEV. These results are consistent with previous studies that demonstrated the ratio of non-responders to LEV in TLE patients and chronically epileptic rats [20,21,22,33]. Basically, the anti-epileptic effects of LEV are relevant to the binding to SV2A [20]. However, LEV also leads to intracellular acidification in neurons, which is coupled with extracellular pH shift [23,34]. Furthermore, LEV inhibits carbonic anhydrase, which leads to extracellular acidification [24,25,26,27]. Since extracellular acidification ceases the seizure activity [7,8,9,10,34], it is likely that extracellular acidic shifts may be one of the anti-epileptic potencies of LEV. Therefore, our findings suggest that the impairment of extracellular acidic shifts may be one of the causes of the low efficacy of LEV in non-responders.

Seizure activity increases the extracellular K^+^ concentration due to K^+^ efflux from neurons during repolarization, accompanied by an initial extracellular alkalinization. Thus, astrocyte-mediated K^+^ buffering plays an important role in ictogenesis and seizure termination to regulate extracellular K^+^ level [7,11,12,35]. Indeed, the low ratio of inward-to-outward K^+^ conductance in astrocytes is reported in the hippocampus of TLE patients [13]. Since extracellular alkalinization potentiates TASK-1-mediated outward K^+^ currents [14,15,36], TASK-1 may participate in a rise of extracellular K^+^ concentration during or following seizure activity. In the present study, TASK-1 expression was increased in reactive CA1 astrocytes of chronically epileptic rats. Furthermore, LEV decreased seizure activity in responders, concomitant with TASK-1 downregulation in CA1 astrocytes. Considering astroglial roles in neuronal hyperexcitability [37,38], it is likely that aberrant elevation of extracellular K^+^ concentration, induced by upregulated TASK-1, may affect seizure activity in the epileptic hippocampus, which would be abolished by LEV-induced extracellular acidification. Conversely, LEV may ameliorate seizure activity by directly regulating astroglial TASK-1 expression independent of extracellular pH shifts, since LEV reduces delayed rectifier K^+^ current [39] and activates renal outer medullary inwardly rectifying K^+^ channel-1 (ROMK1, also known as KCNJ1 or Kir1.1) [40]. The present data also reveal that ML365 effectively diminished TASK-1 expression in CA1 astrocytes and decreased seizure duration in non-responders. In addition, ML365 co-treatment increased the efficacy of LEV. These findings indicate that the inhibition of TASK-1-mediated outwardly K^+^ rectification may overcome the ineffectiveness of LEV in extracellular acidic shifts and/or the direct regulation of TASK-1 expression in non-responders. Therefore, the present data suggest that TASK-1 is a potential therapeutic target for improving the responsiveness to LEV in non-responders.

On the other hand, the localization of TASK-1 in the hippocampus has been still controversial; TASK-1 expression is mainly observed in astrocytes of the hippocampus [16,32], whereas neuronal TASK-1 expression has been also reported [41]. Although the basis remains unclear, this discrepancy may be attributable to the age of animals used in the studies (adult [16,32], the present study vs. young [41]). Indeed, TASK-1 mRNA expression peaks by 7 days postnatal, and then gradually declines by 28 days postnatal in the mouse hippocampus [42]. Conversely, it is plausible that functional levels of TASK-1 expression on neurons would be extremely too low to be detectable with immunohistochemistry. If present on neurons, however, extracellular acidic shifts induced by LEV would result in prolonged depolarization by inhibiting TASK-1-mediated K^+^ efflux from neurons. Furthermore, actions of LEV or ML365 on TASK-1 would increase the excitability of principal neurons and interneurons. Under these conditions, the delayed repolarization would impair the fast-spiking capability of interneurons, and lead to uncontrolled epileptiform discharges in principal neurons due to reduced GABAergic inhibition [43]. Similar to chronically epileptic rats, TASK-1 expression is rarely observed in principal neurons of TLE patients but is predominantly detected in astrocytes [17]. In addition, the massive degeneration of hippocampal neurons (particularly, CA1 pyramidal cells and interneurons) are observed in chronically epileptic rats [28,29]. Therefore, our findings suggest that LEV or ML365 may diminish spontaneous seizures by inhibiting TASK-1 in CA1 astrocytes rather than neurons in chronically epileptic rats.

In the present study, we could not access the underlying mechanisms of the modulation of astroglial TASK-1 expression. However, it is worth considering that serum- and glucocorticoid-inducible kinase (SGK)-mediated signaling pathway may be involved in astroglial TASK-1 regulation. This is because SGK1 activity is lower in chronically epileptic rats as compared to the controls [44], and SGK inhibits TASK-1 current and its surface expression [45]. Further studies are needed to elucidate the role of SGK-mediated signaling pathway in astroglial TASK-1 regulation.

## 5. Conclusions

In the present study, we demonstrated, for the first time, that LEV ameliorated spontaneous seizure activity in responders by reducing the upregulated TASK-1 expression in CA1 astrocytes. Furthermore, ML365 co-treatment improved the efficacy of LEV in non-responders. Therefore, our findings suggest that the dysregulation of TASK-1 function may be one of the causes of refractory seizures to LEV, and TASK-1 inhibition is a potential therapeutic target for improving the responsiveness to LEV in non-responders.

## Figures and Tables

**Table 1:** Primary antibodies used in the present study.

Antigen	Host	Manufacturer (Catalog Number)	Dilution
Glia fibrillary acidic protein (GFAP)	Mouse	Millipore (MAB3402)	1:4000 (IH)
Neuronal nuclear antigen (NeuN)	Guinea pig	Millipore (ABN90P)	1:2000 (IH)
TASK-1	Rabbit	Millipore (AB5250)	1:50 (IH) 1:200 (WB)
β-actin	Mouse	Sigma (A5316)	1:5000 (WB)

IH, immunohistochemistry; WB, Western blot.

## Data Availability

Not applicable.

## Supplementary Materials

The following supporting information can be downloaded at: https://www.mdpi.com/article/10.3390/biomedicines10040787/s1, Figure S1: Full-length gel images of the Western blot data in Figure 3C, Figure 5C and Figure 7C.
