# Supplementary material for: Blockade of TASK-1 Channel Improves the Efficacy of Levetiracetam in Chronically Epileptic Rats
Source: Biomedicines. 2022 Mar 28;10(4):787. doi: 10.3390/biomedicines10040787 (PMC9030960; doi:10.3390/biomedicines10040787)
Supplement: Supplementary file 1 [file biomedicines-10-00787-s001.zip › biomedicines-1618431-supplementary.pdf]

Supplementary information

Blockade of TASK-1 channel in astrocytes improves the efficacy of levetiracetam in chronic epilepsy rats

Ji-Eun Kim*, Tae-Cheon Kang*

Department of Anatomy and Neurobiology and Institute of Epilepsy Research, College of Medicine, Hallym University, Chuncheon 24252, Korea

***** Correspondence: Department of Anatomy and Neurobiology and Institute of Epilepsy Research, College of Medicine, Hallym University, Chuncheon 24252, Korea. E-mail: jieunkim@hallym.ac.kr and tckang@hallym.ac.kr; Tel.: +82-33-248-2522 and +82-33-248-2524; Fax: +82-33-248-2525.

Fig. 3C

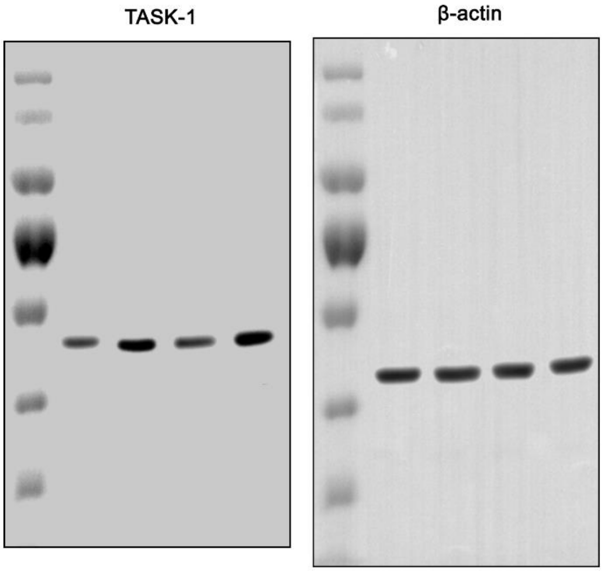

Fig. 5C

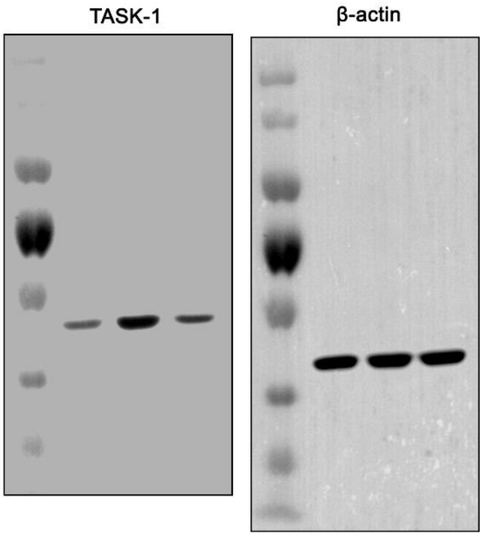

Fig. 7C

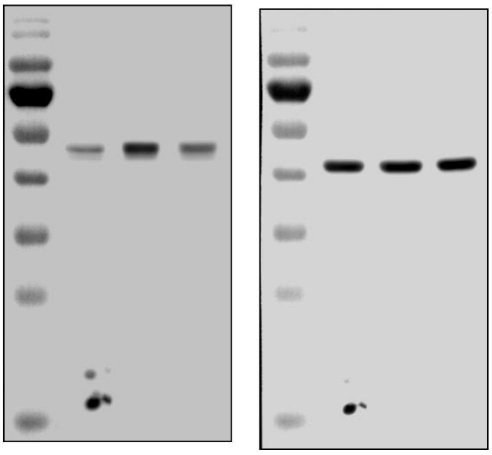

Supplementary Figure S1. Full-length gel images of Western blot data in Figures 3C, 5C and 7C.
